# The role of the left primary motor cortex in apraxia

**DOI:** 10.1186/s42466-024-00359-8

**Published:** 2025-01-09

**Authors:** Ksenia Perlova, Claudia C. Schmidt, Gereon R. Fink, Peter H. Weiss

**Affiliations:** 1 Department of Neurology, Faculty of Medicine and University Hospital Cologne, University of Cologne, Kerpener Str. 62, 50937 Cologne, Germany; 2 Cognitive Neuroscience, Institute of Neuroscience and Medicine (INM-3), Research Centre Jülich, Leo-Brandt-Str. 5, 52425 Jülich, Germany

**Keywords:** Motor cognition, Imitation, Finger gestures, Body-part specificity, Gesture meaning, Limb-kinetic apraxia

## Abstract

**Background:**

Apraxia is a motor-cognitive disorder that primary sensorimotor deficits cannot solely explain. Previous research in stroke patients has focused on damage to the fronto-parietal praxis networks in the left hemisphere (LH) as the cause of apraxic deficits. In contrast, the potential role of the (left) primary motor cortex (M1) has largely been neglected. However, recent brain stimulation and lesion-mapping studies suggest an involvement of left M1 in motor cognitive processes—over and above its role in motor execution. Therefore, this study explored whether the left M1 plays a specific role in apraxia.

**Methods:**

We identified 157 right-handed patients with first-ever unilateral LH stroke in the sub-acute phase (< 90 days post-stroke), for whom apraxia assessments performed with the ipsilesional left hand and lesion maps were available. Utilizing the maximum probability map of Brodmann area 4 (representing M1) provided by the JuBrain Anatomy Toolbox in SPM, patients were subdivided into two groups depending on whether their lesions involved (n = 40) or spared (n = 117) left M1. We applied a mixed model ANCOVA with repeated measures to compare apraxic deficits between the two patient groups, considering the factors “body part” and “gesture meaning”. Furthermore, we explored potential differential effects of the anterior (4a) and posterior (4p) parts of Brodmann area 4 by correlation analyses.

**Results:**

Patients with and without M1 involvement did not differ in age and time post-stroke but in lesion size. When controlling for lesion size, the total apraxia scores did not differ significantly between groups. However, the mixed model ANCOVA showed that LH stroke patients with lesions involving left M1 performed differentially worse when imitating meaningless finger gestures. This effect was primarily driven by lesions affecting Brodmann area 4p.

**Conclusions:**

Even though many current definitions of apraxia disregard a relevant role of (left) M1, the observed differential effect of M1 lesions, specifically involving subarea 4p, on the imitation of meaningless finger gestures in the current sample of LH stroke patients suggests a specific role of left M1 in imitation when high amounts of (motor) attention and sensorimotor integration are required.

**Supplementary Information:**

The online version contains supplementary material available at 10.1186/s42466-024-00359-8.

## Background

Apraxia is a collective term for motor-cognitive deficits that cannot solely be explained by primary sensorimotor deficits, aphasia, or general cognitive decline [1, 2]. The classification of apraxia has been developed from the original distinction between ideational and ideomotor apraxia [3] towards a more descriptive approach, differentiating the body part affected (e.g. bucco-facial, arm, hand, finger), the type of task (imitation, pantomime, actual tool use) and the meaning of gestures (meaningful and meaningless) [4], based on behavioral dissociations in patients with left hemisphere (LH) stroke (for an overview, see e.g. [2, 5].

Unfortunately, the definition of apraxia still depends on exclusion criteria [6, 7]. As basic motor deficits are often considered as one of these exclusion criteria, the primary motor cortex (M1) is rarely discussed as a relevant lesion site causing apraxia. Nevertheless, several lesion-mapping studies have revealed an association between M1 lesions and apraxia [7–18]. These studies detected statistically significant associations of left M1 lesions and apraxic deficits of the ipsilesional left upper limb, thus excluding contralesional paresis as a confounding factor. More precisely, lesions affecting the precentral gyrus have been associated with deficits in the imitation of arm/hand gestures [7, 11], the imitation of (meaningless) finger gestures [9, 12], and the use of novel tools [16].

Notably, these lesion mapping studies did not consider M1 subdivisions. Located in the precentral gyrus, M1 corresponds to Brodmann Area (BA) 4. It can be further divided into two subdivisions (anterior BA 4 (BA 4a) and posterior BA 4 (BA 4p), which differ in cytoarchitecture [19], connectivity, and function. While BA 4a is primarily connected to premotor areas, BA 4p mostly connects to the primary sensory cortex [20]. Furthermore, BA 4p is modulated by attention to motor actions (i.e., motor attention), while BA 4a is not [20].

In this study, we explored the potential role of left M1 lesions in apraxia after LH stroke. First, we examined whether the prevalence of apraxia differed in LH stroke patients whose lesions involved M1 (n = 40) or not (n = 117). Second, we investigated whether M1 lesions were associated with specific apraxic deficits and if so, whether BA 4a or BA 4p lesions differentially impacted higher motor cognition.

## Methods

### Patient sample

In our motor cognition database that comprises the data of our previously published studies on apraxia, neuropsychological and lesion data of 299 patients with a first-ever unilateral ischemic stroke to the left hemisphere were available for analysis. These patients did not suffer from prior neurological diseases affecting the central nervous system or clinically relevant psychiatric diseases. For the current retrospective analyses, we selected those right-handed patients with a subacute LH stroke (i.e., time post-stroke < 90 days), from whom assessments of apraxia (here: Cologne Apraxia Screening (KAS; [21] and imitation tests by Goldenberg for hand positions and finger configurations [22] and aphasia (here: the short form of the Aphasia check-list, ACL-K [23],) and lesion maps were available. The final sample consisted of 157 right-handed LH stroke patients [24] with an age of 62.4 ± 13.6 years (mean ± standard deviation) at the time of stroke and a time post-stroke of 18.5 ± 16.7 days (mean ± standard deviation) at the time of apraxia scoring. Data of these patients have been reported in previous studies on apraxia in left hemisphere stroke [10, 25–29]. Data on motor assessments with the action research arm test (ARAT, [30]) was available for 83 patients with left hemisphere (LH) stroke.

### Lesion data

To differentiate between LH stroke patients with lesions involving and sparing the left M1, we used manually delineated lesion maps (volumes of interest; VOIs) that had been performed on the clinical CT (n = 39) or MRI (n = 118) scans using the MRIcron software package. The lesions had been drawn on axial slices of a T1-weighted template MRI scan (ch2.nii) from the Montreal Neurological Institute (MNI) with a 1 × 1-mm in-plane resolution in steps of 5 mm in MNI space onto the closest matching axial slices of the patients’ cerebral imaging.

**Fig. 1:**
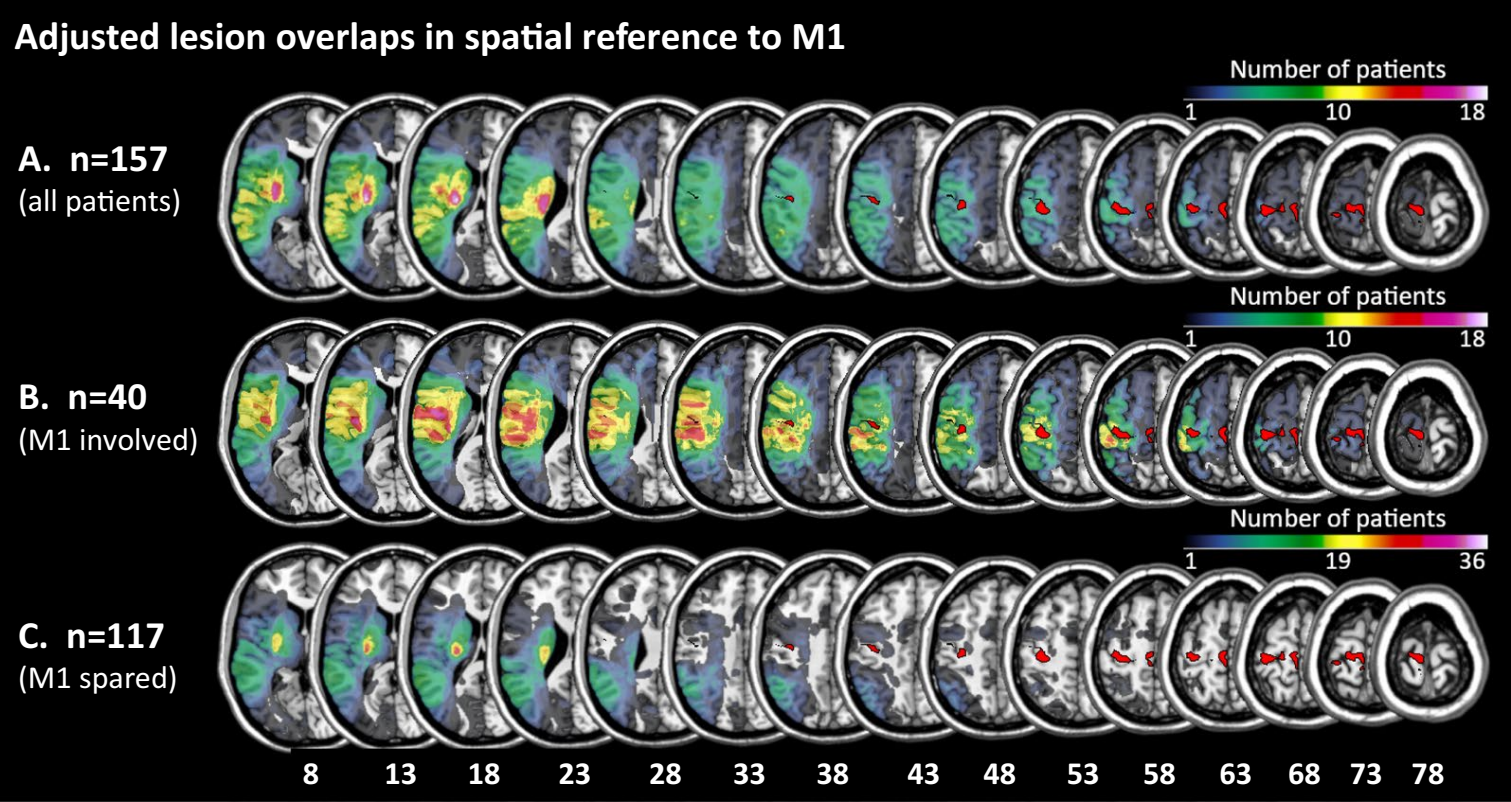
Adjusted lesion overlaps in spatial reference to Area 4 (i.e., the primary motor cortex, M1). **A** Lesion overlay of all left hemisphere (LH) stroke patients (n = 157), **B** Lesion overlay of the LH stroke patients with lesions involving M1 (n = 40), **C** Lesion overlay of the LH patients with lesions sparing M1 (n = 117). Colors represent the number of patients with overlapping lesions at a given position. The figure demonstrates that the lesion overlap in C. does not comprise Area 4 (data taken from the Julich-Brain Atlas [31]

The M1 as the region of interest (ROI) was defined by the combined maximum probability maps of Brodmann areas 4a and 4p of the Julich Brain Anatomy Toolbox, a plugin for SPM [31]. MRIcron (Version 02/09/2019, https://www.nitrc.org/projects/mricron) was used to compute the overlap between the M1-ROI (i.e., BA 4) and the lesion map (volume of interest, VOI) of a given patient. If at least one voxel overlapped, the corresponding LH stroke patient was allocated to the subgroup “M1-lesioned”. If there was no overlap between the M1-ROI and the lesion map, the corresponding LH stroke patient was allocated to the subgroup “M1-spared”. We also computed the percentage of overlapping voxels separately for subareas 4a and 4p. Finally, we calculated the relative lesion size in left M1, expressed as the proportion (i.e., percentage) of overlapping voxels concerning all voxels in BA 4 (6795 voxels), BA 4a (4611 voxels), and BA 4p (2184 voxels). Figure 1 depicts the lesion overlaps of the two groups of LH stroke patients (M1-spared, M1-lesioned) with BA 4. The supplementary Figure S1 depicts BA 4 (Figure S1A) as well as BA 4a and BA4p (Figure S1B) on the standard brain provided by MRIcron.

### Neuropsychological assessment

#### Cologne apraxia screening (KAS)

The Cologne Apraxia Screening (Kölner Apraxie Screening; KAS) evaluates the performance of pantomimes and the imitation of bucco-facial and arm/hand gestures [21] performed with the ipsilesional left hand in case of left hemisphere stroke.

The test comprises 20 items with a total score of 80 points. The four subtests consist of five items each: (1) pantomiming the use of bucco-facial related tools/objects, (2) pantomiming the use of arm/hand related tools/objects, (3) imitation of bucco-facial gestures, and (4) imitation of arm/hand gestures. The two imitation subtests include three meaningless (ML) and two meaningful (MF) gestures for each body part. Each item is scored with a maximum of four points. For a detailed description of the test, see Schmidt et al. [7]. Patients are defined as apraxic, if they score less than 77 points on the KAS.

#### Goldenberg imitation tests

The imitation of hand and finger gestures was tested by the mirror-like demonstration of ten meaningless hand positions and ten finger configurations [22]. Of the ten finger gestures, two configurations are considered clearly ML and three configurations are judged clearly MF [32]. The imitation of each of the 20 gestures is scored with 0–2 points. Patients imitated the gestures with their ipsilesional left hand and fingers. They were defined as apraxic when they scored less than 18 (of 20) points in the hand imitation test or less than 17 (of 20) points in the finger imitation test.

Overall, patients were considered apraxic if they scored below cut-off in at least one of the three aforementioned apraxia tests (KAS, finger gesture imitation, hand gesture imitation).

All patients with left hemisphere stroke performed the three apraxia assessments with their ipsilesional left hand and fingers. This approach is typically chosen to minimize the effects of motor deficits caused by contralesional paresis. However, ipsilesional motor deficits of the upper limb have been described for left hemisphere and right hemisphere stroke [33].

#### Aphasia check-list-short form (ACL-K)

Language skills were assessed using the short form of the aphasia check list (ACL-K), which encompasses four sections: reading-aloud, auditory comprehension, verbal fluency, and a rating of the patient’s verbal communication skills by the examiner [23]. Patients are classified as aphasic if they score below the cut-off of 33 points (maximum score: 40).

### Statistical analysis

The statistical analyses were performed using IBM SPSS Statistics (Statistical Package for the Social Sciences, version 29.0.0.0, SPP Inc., Chicago, Illinois, USA) and JASP (JASP Team (2023), version 0.17.1). We applied a significance level of *p* < 0.05 for all analyses.

To characterize the two patient groups (M1-lesioned vs. M1-spared), we calculated the means for lesion size (number of lesioned voxels), ACL-K score, age at time of stroke, and time post-stroke, and checked for group differences using t-tests. We further compared the sex distribution in the groups using a Chi-Square-test. For further analyses, we used lesion size and the ACL-K score as covariates.

The performances in the three apraxia assessments were converted into percentage scores to allow direct comparison of the differently scaled tests. Applying an ANCOVA, we compared the performance of the M1-lesioned and M1-spared groups in the three apraxia assessments with lesion size and ACL-K score as covariates.

A Chi^2^ test was performed to determine, whether M1-lesioned patients were diagnosed with apraxia more frequently than the M1-spared group.

Further, three repeated measures ANCOVAs with lesion size and ACL-K score as covariates were performed for different apraxia scores to evaluate the putative effects of action domain, body part, and gesture meaning. In all three ANCOVAs, M1-involvement (M1-spared, M1-lesioned) was the between-subject factor. The first 2 × 2 ANCOVA evaluated potential group differences in the scores of the KAS subtests on pantomiming and imitation by including the within-factor ACTION DOMAIN. The second 2 × 2 ANCOVA evaluated potential group differences in the scores of the two KAS pantomime subtests by including the within-factor BODY PART (bucco-facial versus arm/hand). The third 2 × 3 × 2 ANCOVA evaluated potential differential group differences in the scores of the imitation tests. To account for the different body parts used for imitation, the within-factor BODY PART (bucco-facial [KAS], arm/hand [KAS], finger [Goldenberg]) was included. Besides, the within-factor gesture MEANING (meaningful [MF] gestures versus meaningless [ML] gestures) was used to account for the meaning of the to be imitated gestures. Where appropriate, degrees of freedom were Greenhouse–Geisser corrected.

Lastly, we performed non-parametric correlations (spearman rho) of the scores of meaningless and meaningful finger configurations with the lesion overlap of BA 4, BA 4a, and BA 4p, while controlling for lesion size and total ACL-K score.

## Results

### Sample characteristics

Based on the overlap of voxels between our patient’s VOIs and the M1-ROI, 40 patients were categorized as having lesions involving the left M1 (M1-lesioned group) and 117 patients with lesions sparing the left M1 (M1-spared group). The lesion size was significantly higher [t(41) = − 4.2, *p* < 0.001] and the ACL-K score significantly lower [t(58) = 2.6, *p* < 0.05] in the M1-lesioned group, while age at the time of stroke did not differ significantly between the groups [t(155) = 1.70, *p* = 0.092] and neither did the time between stroke and neuropsychological examination [t(57) = − 2.0, *p* = 0.052; see Table 1]. Gender was distributed equally in the two groups [χ^2^(1) = 2.33, *p* = 0.136].

**Table 1:** **-** Characterizing patient subgroups based on involvement of left M1

	Patients with lesions involving left M1 (n = 40)	Patients with lesions sparing left M1 (n = 117)	Differences between groups
Lesion size (voxels)	11,977.2 ± 13,735.2	2715.9 ± 3969.9	*p* < 0.001*
ACL-K score	23.7 ± 12.0	29.1 ± 9.9	*p* < 0.05*
Age (years)	59.3 ± 12.0	63.5 ± 14.0	n.s. (*p* = 0.092)
Time post-stroke (days)	23.5 ± 19.3	16.8 ± 15.5	n.s. (*p* = 0.052)
Sex (w/m)	11/29	48/69	n.s. (*p* = 0.136)

ACL-K = Aphasia check-list-short version. Given are means and standard deviations (SD; in parentheses). Significant correlations at a *p*-value of < 0.05 are marked with an asterisk and bold print

Consistent with the observation that the ARAT does not have a high sensitivity for detecting subtle motor impairments [34], only seven of the 83 stroke patients (8.4%) did not achieve the full score in the ARAT, when tested with their ipsilesional left hand. Notably, all seven patients lost their points in the ARAT-subtest “pinch” assessing the grasping of small items with a pinch grip. When tested statistically, there were no significant differences in the ARAT scores for the left ipsilesional hand (Mann–Whitney-U-test, U = 620.5, *p* = 0.164) between the LH stroke patients with lesions involving or sparing left M1. Moreover, the ARAT scores for the ipsilesional, left hand did not significantly differ between the LH stroke patients with and without apraxia (Mann–Whitney-U-test, U = 777.0, *p* = 0.139) and these scores did not correlate with the scores of the three apraxia assessments (KAS: Spearman’s *ρ* = 0.117, *p* = 0.293; Goldenberg hand imitation: Spearman’s *ρ* = 0.108, *p* = 0.330; Goldenberg finger imitation: Spearman’s *ρ* = − 0.108, *p* = 0.330). Finally, the ARAT scores for the ipsilesional left hand did not correlate with the performance of imitating meaningless finger gestures with the ipsilesional left hand (Spearman’s *ρ* = 0.057, *p* = 0.610). These results are—at least in part—due to the ceiling effects observed in the ARAT. Therefore, forthcoming studies on stroke-related apraxic deficits of the ipsilesional hand should consider including a comprehensive assessment of fine motor skills, e.g., the Pegboard test.

### Apraxia assessments

The overall performance in the apraxia tests did not differ significantly between the two subgroups. For the KAS, there was a significant effect of lesion size [F(1,153) = 8.49, *p* < 0.01] and ACL-K score [F(1,153) = 34.75, *p* < 0.001] on performance. M1-involvement did not significantly affect the KAS total score after controlling for the two covariates [F(1,153) = 0.01, *p* = 0.908]. Lesion size had no significant effect on the hand imitation score by Goldenberg [F(1,153) = 0.02, *p* = 0.893], while ACL-K had a significant effect [F(1,153) = 31.30, *p* < 0.001]. M1-involvement had no significant impact on the hand imitation score after controlling for the two covariates [F(1,153) = 0.51, *p* = 0.477]. Lesion size did not have a significant effect on the finger imitation score [F(1,153) = 1.59, *p* = 0.210], in contrast to the ACL-K [F(1,153) = 11.33, *p* < 0.001]. After controlling for the covariates, M1-involvement did not significantly influence the finger imitation score [F(1,153) = 3.61, *p* = 0.059]. Apraxia in general was similarly frequent in the two groups [χ^2^(1) = 1.56, *p* = 0.212].

### Effects of action domain, body part, and gesture meaning on apraxic deficits

#### Differential effect of action domain

The ANCOVA with the KAS scores revealed no significant main effects for “Action domain” [F(1,153) = 0.279, *p* = 0.598], M1-involvement [F(1,153) = 0.014, *p* = 0.908], and the interaction “Action domain” x M1-involvement [F(1,153) = 0.512, *p* = 0.476]. Therefore, in the current sample of LH stroke patients, performance was similar for pantomime and imitation tasks, and M1-involvement did not have a (differential) effect on the two tasks.

#### Differential effect of body part in pantomime

The ANCOVA for the KAS pantomime scores revealed a significant main effect for “Body part” [F(1,153) = 4.495, *p* = 0.036]. The current LH stroke patients performed better in the bucco-facial pantomime subtest (91.5% ± 1.4%, marginal mean ± SE) than in the arm/hand pantomime subtest (89.5% ± 1.5%, marginal mean ± SE) of the KAS. The main effect for M1 was not significant [F(1,153) = 0.153, *p* = 0.696] and neither was the interaction for “Body part” x M1 [F(1,153) = 0.034, *p* = 0.854].

#### Differential effects of body part and meaning in imitation

The ANCOVA for the scores in the Goldenberg finger imitation test and the KAS subtests imitation of bucco-facial gestures and imitation of arm/hand gestures revealed no significant main effects for “Body part” [F(2,306) = 1.854, *p* = 0.158] and M1-involvement [F(1,153) = 1.536, *p* = 0.217]. However, the main effect for “Meaning” was significant [F(1,153) = 5.372, *p* < 0.05]. The pairwise comparison (Bonferroni-corrected) revealed that LH stroke patients performed significantly worse for meaningless compared to meaningful gestures (mean diff. = 6.8%, SE = 1.3, *p* < 0.001).

The “Body part x Meaning” interaction was significant [F(2,306) = 6.079, *p* = 0.003]. Notably, meaning modulated only upper-limb gestures. Specifically, meaningful finger gestures (mean diff. = 18.362%, SE = 2.295, *p* < 0.001) and meaningful hand gestures (mean diff. = 4.686%, SE = 1.947, *p* < 0.05) were better imitated than their meaningless counterparts, while the imitation of bucco-facial gestures was not significantly modulated by gesture meaning (mean diff. = 2.610%, SE = 1.712, *p* = 0.130).

**Fig. 2:**
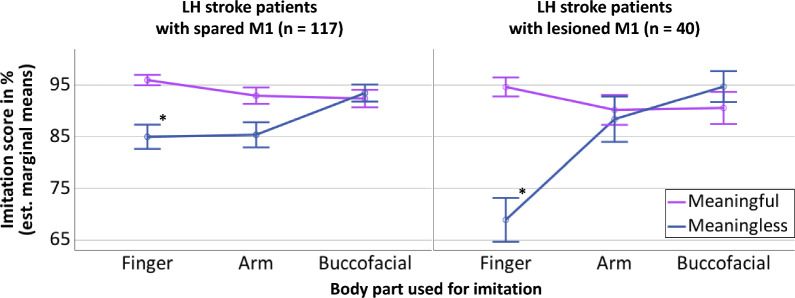
Differential effect of M1-lesions on meaningless finger gestures. Graphical depiction of the significant three-way interaction for the mean imitation scores revealed by the ANCOVA with the between subject-factor M1 involvement (M1 intact—left, M1 lesioned—right) and the within-subject factors meaning (meaningful gestures—purple lines, meaningless gestures—blue lines) and body part used for imitation (finger, arm, bucco-facial). The asterisks highlight the differential impairment of LH stroke patients with lesions involving M1 when imitating meaningless finger gestures

The three-way interaction for “Body part” x “Meaning” x M1 was also significant [F(2,306) = 7.140, *p* < 0.001]. As the current study focused on the involvement of M1 in apraxia, we performed pairwise comparisons of the two patient groups (M1-spared, M1-involved), e.g., the imitation score for meaningful/meaningless arm gestures of the LH stroke patients with M1 spared versus the imitation score for meaningful/meaningless arm gestures of the LH stroke patients with lesions involving M1. Here, only the pairwise comparison of meaningless finger gestures was significant [F(1,153) = 9.974, *p* = 0.002]. Meaningless finger gestures were performed worse by patients with lesions involving the left M1 (mean diff. = 16.044%, SE = 5.080; see Fig. 2, the differential effects for meaningless finger gestures are marked with asterisks).

### Correlation of lesion load in BA 4 with imitation scores

To further examine the relationship between apraxic deficits in finger imitation and lesions in M1 (i.e., Brodmann area 4) as well as lesions in the two subareas of M1 (i.e., Brodmann areas 4a and 4p), we performed non-parametric correlation analysis with the mean imitation scores of the Goldenberg finger imitation test and the lesion loads in area 4 as well as subareas 4a and 4p (operationalized as the number of affected voxels in a given area, see Table 2). The lesion load in Area 4p correlated significantly with the mean imitation scores, when the MF and ML finger gestures of the Goldenberg imitation test were combined [rho = − 0.339, *p* = 0.037]. Importantly, the mean scores for imitating meaningless finger gestures also correlated significantly with the lesion load in Area 4p [rho = − 0.323, *p* = 0.048], while the mean imitation score for meaningful finger gestures did not. The lesion load in area 4a as well as the lesion load in area 4 (i.e., areas 4a and 4p combined) did not correlate significantly with any finger imitation score.

**Table 2:** Correlation of lesion loads in Brodmann areas 4, 4a, 4p with finger gesture imitation scores

	Area 4	Area 4a	Area 4p
Imitation of the ML and MF finger gestures combined	rho = − 0.244,	rho = − 0.113,	**rho = − 0.339,**
	*p* = 0.139, n.s	*p* = 0.499, n.s	*p* = 0.037*
Imitation of ML finger gestures	rho = − 0.233,	rho = − 0.111,	**rho = − 0.323,**
	*p* = 0.160, n.s	*p* = 0.507, n.s	*p* = 0.048*
Imitation of MF finger gestures	rho = − 0.101,	rho = − 0.004,	rho = − 0.188,
	*p* = 0.544, n.s	*p* = 0.979, n.s	*p* = 0.257, n.s

Non-parametric correlation analyses (Spearman rho) of the lesion loads in Brodmann areas 4, 4a, and 4p with the imitation score for ML and MF finger gestures combined as well as the separate imitation scores for meaningless (ML) and meaningful (MF) finger gestures. The overall lesion size and the ACL-K score were used as covariates. The analysis was performed selectively for patients with lesions involving the primary motor cortex (M1, n = 40). Significant correlations at a *p*-value of < 0.05 are marked with an asterisk and bold print

## Discussion

This study elucidated the role of the left primary motor cortex (M1) in cognitive-motor functions (i.e., praxis) by analyzing the association of lesions affecting the left M1 with apraxic deficits. When comparing two groups of patients with left hemisphere (LH) strokes that either involved (n = 40) or spared the left M1 (n = 117), there was no group difference in the overall apraxia scores or the incidence of apraxia. In contrast, when considering the body part used for imitation and gesture meaning, the analyses revealed a significant differential impairment of the LH stroke patients with lesions involving M1 when imitating meaningless finger gestures. Moreover, the meaningless finger gesture imitation scores correlated negatively with the lesion load in Area 4p. Thus, LH stroke patients whose lesions affected more of Area 4p performed worse when imitating meaningless finger gestures. Although most definitions of apraxia dispute an involvement of M1 in apraxia, the current findings strongly suggest a specific role of the left M1 in imitating meaningless finger gestures that put high demands on (motor) attention and sensorimotor integration.

That the meaning of a gesture affects imitation performance in patients with LH damage and (limb) apraxia has already been reported [32, 35–39]. Based on the observed dissociations between imitating meaningless (ML) versus meaningful (MF) gestures, the two-route model has been proposed. Here, two distinct routes, direct and indirect, support the imitation of ML and MF gestures, respectively [38]. Since the indirect route can process meaningful gestures by retrieving pre-existing motor representations, even when the visuomotor transformation mechanisms of the direct route are disturbed, the most frequently reported dissociations relate to preserved imitation of meaningful gestures and impaired imitation of meaningless gestures [9]. Moreover, lesions to the left precentral gyrus (i.e., the gyrus containing M1) have been linked to producing meaningless gestures [9]. Consistent with these previous findings, the current LH stroke patients with lesions involving left M1 showed a selective deficit when imitating meaningless finger gestures.

The differential apraxic deficit in imitating meaningless finger gestures is reminiscent of the concept of limb-kinetic apraxia, as proposed by Liepmann, which comprises deficits in dexterous movements impairing the proficiency in “precise and independent but coordinated finger movements” [40]. Although limb-kinetic apraxia has initially been described as a deficit of the contralesional hand following lesions to the sensorimotor cortex, Liepmann already noted additional, although milder deficits to the ipsilesional hand for left-hemispheric lesions, which he called dyspraxia [41], see also [42]. Following studies elaborated on a left-hemispheric involvement in dexterous movement of the ipsilateral left hand, while right hemisphere lesions did not appear to impact dexterity of the ipsilateral right hand [40, 43, 44].

The current analysis of the gesture imitation scores also revealed that gesture meaning had hardly any effect in both patient groups on the imitation of bucco-facial gestures. This finding is consistent with a recent study in LH stroke patients revealing an effector-specific effect of gesture meaning on imitation performance for arm/hand gestures but not for bucco-facial gestures [36].

Brodmann Area (BA) 4 constitutes M1. Importantly, BA 4 comprises two subareas, namely anterior BA 4 (area 4a) and posterior BA 4 (area 4p). Concerning finger gesture imitation, we found a moderate correlation of the lesion load in area 4p with apraxic deficits when imitating meaningless finger gestures but not when imitating meaningful finger gestures. In contrast, the lesion load in area 4a did not correlate significantly with any imitation scores. While area 4a is primarily connected to premotor areas, area 4p mainly connects to the primary sensory cortex [20]. Unlike BA 4a, BA 4p is modulated by attention to motor actions [20]. It is conceivable that imitating meaningless finger gestures puts more demands on motor attention than imitating meaningful finger gestures, especially since meaningless finger gestures are considered more difficult to be imitated [32]. In a similar vein, the precise replication of the meaningless finger gestures may require more sensory feedback during the imitation to adjust the unfamiliar finger configurations according to the template. Such feedback processing is likely to happen in the part of M1 connected to the sensory cortex, i.e., area 4p. Taken together, the differential apraxic imitation deficits of LH stroke patients with lesions involving left M1 when imitating ML finger gestures may result from impaired motor attention and/or sensory integration processes supported by left M1. The data suggest that forthcoming definitions of apraxia should consider the role of left M1 in (imitation) apraxia.

This is especially warranted, since a recent study by Gordon and colleagues suggests that the classical somatotopic representation of the body in M1 is interspaced by three zones (inter-effector regions) in which complex actions are represented and which are interconnected by the "somato-cognitive action network" (SCAN, [45]). The findings of Gordon and colleagues converge with our results showing that complex actions, like imitating meaningless finger gestures (executed with the ipsilateral/ipsilesional hand), are impaired in (LH stroke) patients suffering from lesions affecting the left M1. Moreover, the functionally defined middle inter-effector region of the SCAN (but not the superior and inferior inter-effector regions) has an overlap with the cyto-architectonically defined M1-subregion area 4p. Notably, there is no relevant overlap of the inter-effector regions described by Gordon and colleagues with area 4a. This pattern of overlap within left M1 is consistent with our finding that the lesion load in area 4p (and not the lesion load in area 4a) is significantly associated with apraxic deficits when imitating meaningless finger gestures. Together, these findings indicate that M1 is not simply a motor output area. As a future perspective, it would be interesting to specifically investigate how the SCAN relates to apraxia, e.g., by connectivity analyses based on resting state functional MRI in apraxic patients.

Some limitations should be considered. As usual, the patients with LH stroke performed the (limb) apraxia assessments with their ipsilesional left hand. Testing the ipsilesional hand should minimize the effects of fundamental motor deficits (e.g., deficits in grip strength and movement speed on the apraxia test scores [34]. However, also the ipsilesional hand is subject to subtle motor deficits impairing dexterity [46]. Since meaningless finger gestures are considered especially difficult to imitate [32], subtle dexterity deficits of the ipsilesional hand could have contributed to the selective deficit in imitating meaningless finger gestures in LH stroke patients with lesions involving left M1.

Since no patients with right hemisphere (RH) lesions were included in the current study, we cannot infer the potential role of the right M1 in apraxia, particularly concerning apraxic deficits in imitating (ML) finger gestures. However, this is especially warranted since previous studies highlight the involvement of the right hemisphere in the imitation of finger configurations [47, 48]. Moreover, these studies clearly showed an association between finger imitation deficits and neglect in patients with RH stroke [47, 49] and suggest that centrally located lesions in the RH instead induce apraxic deficits in finger gesture imitation than in hand gesture imitation [49]. However, none of the previous studies investigating RH stroke patients differentiated between the imitation of MF and ML finger configurations.

## Conclusion

Current apraxia definitions implicitly deny the role of the left M1 in apraxia. However, several previous studies employing lesion mapping have mentioned or displayed left M1 lesions in association with apraxic deficits without further interpretation. Here, we provide the first systematic investigation on the involvement of left M1 in apraxia, revealing a specific apraxic imitation deficit for meaningless finger gestures in LH stroke patients with lesions involving left M1. This differential apraxic imitation deficit appears to be driven by the lesion load in left area 4p, i.e., the part of left M1 modulated by motor attention and supporting sensory integration.

## Supplementary Information

Additional file1. Figure S1 – Brodmann Area 4 and its anterior and posterior subareas displayed on the standard brain. A. Brodmann Area 4 (6795 voxels, red); B. Brodmann Areas 4a (4611 voxels, green) and 4p (2184 voxels, blue). The regions of interest (ROIs), taken from the Julich-Brain Atlas (Amunts et al., 2021), are displayed on the standard brain provided by MRIcron.

## Data Availability

The datasets used and/or analysed during the current study are available from the corresponding author on reasonable request.

## Abbreviations

ACL-K: Aphasia check-list-short form
ARAT: Action research arm test
ANCOVA: Analysis of covariance
BA: Brodmann area
BA 4a: Anterior part of Brodmann area 4
BA 4p: Posterior part of Brodmann area 4
CT: Computed tomography
KAS: Cologne apraxia screening
LH: Left hemisphere
M1: Primary motor cortex
MF: Meaningful
ML: Meaningless
MRI: Magnetic resonance imaging
ROI: Region of interest
RH: Right hemisphere
SCAN: Somato-cognitive action network
SPM: Statistical parametric mapping
SPSS: Statistical package for the social sciences
VOI: Volume of interest

## References

[1] Rothi, L. J. G., & Heilman, K. M. (1997). *Apraxia: The NEUROPSYCHOLOGY OF ACTION*. Psychology Press. 10.4324/9781315804545

[2] Schmidt, C. C., & Weiss, P. H. (2022). The cognitive neuroscience of apraxia. In S. Della Sala (Ed.), *Encyclopedia of behavioral neuroscience* (Vol. 2, pp. 668–677). London: Elsevier. 10.1016/b978-0-12-819641-0.00143-2

[3] Liepmann, H. (1913). Motorische Aphasie und Apraxie. *Monatsschrift für Psychiatrie und Neurologie,**34*, 485–494. 10.1159/000203164

[4] Goldenberg, G. (2014). Apraxia—The cognitive side of motor control. *Cortex,**57*, 270–274. 10.1016/j.cortex.2013.07.016 24144066 10.1016/j.cortex.2013.07.016

[5] Bieńkiewicz, M. M. N., Brandi, M. L., Goldenberg, G., Hughes, C. M. L., & Hermsdörfer, J. (2014). The tool in the brain: apraxia in ADL. Behavioral and neurological correlates of apraxia in daily living. *Frontiers in Psychology*. 10.3389/fpsyg.2014.00353 24795685 10.3389/fpsyg.2014.00353 PMC4005934

[6] Petreska, B., Adriani, M., Blanke, O., & Billard, A. G. (2007). Apraxia: A review. *Progress in Brain Research,**164*, 61–83. 10.1016/S0079-6123(07)64004-7 17920426 10.1016/S0079-6123(07)64004-7

[7] Schmidt, C. C., Achilles, E. I. S., Fink, G. R., & Weiss, P. H. (2022). Distinct cognitive components and their neural substrates underlying praxis and language deficits following left hemisphere stroke. *Cortex,**146*, 200–215. 10.1016/j.cortex.2021.11.004 34896806 10.1016/j.cortex.2021.11.004

[8] Achilles, E. I. S., Weiss, P. H., Fink, G. R., Binder, E., Price, C. J., & Hope, T. M. H. (2017). Using multi-level Bayesian lesion-symptom mapping to probe the body-part-specificity of gesture imitation skills. *NeuroImage,**161*(8), 94–103. 28822751 10.1016/j.neuroimage.2017.08.036 PMC5692920

[9] Achilles, E. I. S., Ballweg, C. S., Niessen, E., Kusch, M., Ant, J. M., Fink, G. R., & Weiss, P. H. (2019). Neural correlates of differential finger gesture imitation deficits in left hemisphere stroke. *NeuroImage: Clinical*. 10.1016/j.nicl.2019.101915 31491825 10.1016/j.nicl.2019.101915 PMC6627029

[10] Binder, E., Dovern, A., Hesse, M. D., Ebke, M., Karbe, H., Saliger, J., Fink, G. R., & Weiss, P. H. (2017). Lesion evidence for a human mirror neuron system. *Cortex,**90*, 125–137. 10.1016/j.cortex.2017.02.008 28391066 10.1016/j.cortex.2017.02.008

[11] Buxbaum, L. J., Shapiro, A. D., & Coslett, H. B. (2014). Critical brain regions for tool-related and imitative actions: A componential analysis. *Brain,**137*(7), 1971–1985. 24776969 10.1093/brain/awu111 PMC4065019

[12] Goldenberg, G., & Karnath, H. O. (2006). The neural basis of imitation is body part specific. *The Journal of Neuroscience: The Official Journal of the Society for Neuroscience,**26*(23), 6282–6287. 10.1523/JNEUROSCI.0638-06.2006 16763035 10.1523/JNEUROSCI.0638-06.2006 PMC6675202

[13] Nobusako, S., Ishibashi, R., Takamura, Y., Oda, E., Tanigashira, Y., Kouno, M., Tominaga, T., Ishibashi, Y., Okuno, H., & Nobusako, K. (2018). Distortion of visuo-motor temporal integration in apraxia: Evidence from delayed visual feedback detection tasks and voxel-based lesion-symptom mapping. *Frontiers in Neurology,**9*(8), 1–23. 30210434 10.3389/fneur.2018.00709 PMC6119712

[14] Randerath, J., Goldenberg, G., Spijkers, W., Li, Y., & Hermsdörfer, J. (2010). Different left brain regions are essential for grasping a tool compared with its subsequent use. *NeuroImage,**53*(1), 171–180. 10.1016/j.neuroimage.2010.06.038 20600986 10.1016/j.neuroimage.2010.06.038

[15] Scandola, M., Gobbetto, V., Bertagnoli, S., Bulgarelli, C., Canzano, L., Aglioti, S. M., & Moro, V. (2021). Gesture errors in left and right hemisphere damaged patients: A behavioural and anatomical study. *Neuropsychologia,**162*(3), 108027. 34560143 10.1016/j.neuropsychologia.2021.108027

[16] Stoll, S. E. M., Finkel, L., Buchmann, I., Hassa, T., Spiteri, S., Liepert, J., & Randerath, J. (2022). 100 years after Liepmann-Lesion correlates of diminished selection and application of familiar versus novel tools. *Cortex,**146*, 1–23. 10.1016/j.cortex.2021.10.002 34801831 10.1016/j.cortex.2021.10.002

[17] Watson, C. E., & Buxbaum, L. J. (2015). A distributed network critical for selecting among tool-directed actions. *Cortex,**65*, 65–82. 10.1016/j.cortex.2015.01.007 25681649 10.1016/j.cortex.2015.01.007 PMC4385438

[18] Weiss, P. H., Ubben, S. D., Kaesberg, S., Kalbe, E., Kessler, J., Liebig, T., & Fink, G. R. (2016). Where language meets meaningful action: A combined behavior and lesion analysis of aphasia and apraxia. *Brain Structure & Function,**221*(1), 563–576. 10.1007/s00429-014-0925-3 25352157 10.1007/s00429-014-0925-3

[19] Geyer, S., Ledberg, A., Schleicher, A., Kinomura, S., Schormann, T., Burgel, U., Klingberg, T., Larsson, J., Zilles, K., & Roland, P. E. (1996). Two different areas within the primary motor cortex of man. *Nature,**382*(6594), 805–807. 10.1038/382805A0 8752272 10.1038/382805a0

[20] Binkofski, F., Fink, G. R., Geyer, S., Buccino, G., Gruber, O., Shah, N. J., Taylor, J. G., Seitz, R. J., Zilles, K., & Freund, H. J. (2002). Neural activity in human primary motor cortex areas 4a and 4p is modulated differentially by attention to action. *Journal of Neurophysiology,**88*(1), 514–519. 10.1152/jn.2002.88.1.514 12091573 10.1152/jn.2002.88.1.514

[21] Weiss, P. H., Kalbe, E., Scherer, A., Binder, E., Kessler, J., & Fink, G. R. (2013). *Das Kölner Apraxie-Screening (KAS)*. Hogrefe. 10.1055/s-0042-115843 27788554

[22] Goldenberg, G. (1996). Defective imitation of gestures in patients with damage in the left or right hemispheres. *Journal of Neurology Neurosurgery and Psychiatry,**61*(2), 176–180. 10.1136/jnnp.61.2.176 8708686 10.1136/jnnp.61.2.176 PMC1073992

[23] Kalbe, E., Reinhold, N., Brand, M., Markowitsch, H. J., & Kessler, J. (2005). A new test battery to assess aphasic disturbances and associated cognitive dysfunctions—German normative data on the aphasia check list. *Journal of Clinical and Experimental Neuropsychology,**27*, 779–794. 10.1080/13803390490918273 16183613 10.1080/13803390490918273

[24] Oldfield, R. C. (1971). The assessment and analysis of handedness: The Edinburgh inventory. *Neuropsychologia,**9*(1), 97–113. 10.1016/0028-3932(71)90067-4 5146491 10.1016/0028-3932(71)90067-4

[25] Ant, J. M., Niessen, E., Achilles, E. I. S., Saliger, J., Karbe, H., Weiss, P. H., & Fink, G. R. (2019). Anodal tDCS over left parietal cortex expedites recovery from stroke-induced apraxic imitation deficits: A pilot study. *Neurological Research and Practice,**1*, 38. 10.1186/s42466-019-0042-0 33324903 10.1186/s42466-019-0042-0 PMC7650120

[26] Dafsari, H. S., Dovern, A., Fink, G. R., & Weiss, P. H. (2019). Deficient body structural description contributes to apraxic end-position errors in imitation. *Neuropsychologia*. 10.1016/j.neuropsychologia.2019.107150 31369744 10.1016/j.neuropsychologia.2019.107150

[27] Dovern, A., Fink, G. R., Timpert, D. C., Saliger, J., Karbe, H., Weiss, P. H., & Koch, I. (2016). Timing matters? Learning of complex spatiotemporal sequences in left-hemisphere stroke patients. *Journal of Cognitive Neuroscience,**28*(2), 223–236. 10.1162/jocn_a_00890 26439271 10.1162/jocn_a_00890

[28] Kusch, M., Gillessen, S., Saliger, J., Karbe, H., Binder, E., Fink, G. R., Vossel, S., & Weiss, P. H. (2018). Reduced awareness for apraxic deficits in left hemisphere stroke. *Neuropsychology,**32*(4), 509–515. 10.1037/neu0000451 29672072 10.1037/neu0000451

[29] Kusch, M., Schmidt, C. C., Goden, L., Tscherpel, C., Stahl, J., Saliger, J., Karbe, H., Fink, G. R., & Weiss, P. H. (2018). Recovery from apraxic deficits and its neural correlate. *Restorative Neurology and Neuroscience,**36*(6), 669–678. 10.3233/rnn-180815 30282379 10.3233/RNN-180815

[30] Lyle, R. C. (1981). A performance test for assessment of upper limb function in physical rehabilitation treatment and research. *International Journal of Rehabilitation Research,**4*(4), 483–492. 7333761 10.1097/00004356-198112000-00001

[31] Amunts, K., Mohlberg, H., Bludau, S., Caspers, S., Brandstretter, A., Eickhoff, S. B., Pieperhoff, P., & Dickscheid, T. (2021). Julich-Brain Atlas-whole-brain collections of cytoarchitectonic probabilistic maps (v2.9). *EBRAINS*. 10.25493/46HK-XMM

[32] Achilles, E. I. S., Fink, G. R., Fischer, M. H., Dovern, A., Held, A., Timpert, D. C., Schroeter, C., Schuetz, K., Kloetzsch, C., & Weiss, P. H. (2016). Effect of meaning on apraxic finger imitation deficits. *Neuropsychologia,**82*, 74–83. 26721762 10.1016/j.neuropsychologia.2015.12.022

[33] Schaefer, S. Y., Haaland, K. Y., & Sainburg, R. L. (2007). Ipsilesional motor deficits following stroke reflect hemispheric specializations for movement control. *Brain,**130*(8), 2146–2158. 10.1093/brain/awm145 17626039 10.1093/brain/awm145 PMC3769213

[34] Wunderle, V., Kuzu, T. D., Tscherpel, C., Fink, G. R., Grefkes, C., & Weiss, P. H. (2024). Age- and sex-related changes in motor functions: A comprehensive assessment and component analysis. *Frontiers Aging Neuroscience,**16*, 1368052. 10.3389/fnagi.2024.1368052 10.3389/fnagi.2024.1368052 PMC11133706 38813530

[35] Cubelli, R., Marchetti, C., Boscolo, G., & Della Sala, S. (2000). Cognition in action: Testing a model of limb apraxia. *Brain and Cognition,**44*(2), 144–165. 11041987 10.1006/brcg.2000.1226

[36] Kleineberg, N. N., Schmidt, C. C., Richter, M. K., Bolte, K., Schloss, N., Fink, G. R., & Weiss, P. H. (2023). Gesture meaning modulates the neural correlates of effector-specific imitation deficits in left hemisphere stroke. *NeuroImage: Clinical,**37*, 103331. 36716655 10.1016/j.nicl.2023.103331 PMC9900453

[37] Rumiati, R. I., & Tessari, A. (2002). Imitation of novel and well-known actions: The role of short-term memory. *Experimental Brain Research,**142*(3), 425–433. 10.1007/s00221-001-0956-x 11819052 10.1007/s00221-001-0956-x

[38] Tessari, A., Canessa, N., Ukmar, M., & Rumiati, R. I. (2007). Neuropsychological evidence for a strategic control of multiple routes in imitation. *Brain,**130*, 1111–1126. 17293356 10.1093/brain/awm003

[39] Tessari, A., Mengotti, P., Faccioli, L., Tuozzi, G., Boscarato, S., Taricco, M., & Rumiati, R. I. (2021). Effect of body-part specificity and meaning in gesture imitation in left hemisphere stroke patients. *Neuropsychologia,**151*, 107720. 33309676 10.1016/j.neuropsychologia.2020.107720

[40] Heilman, K. M. (2020). Hugo Liepmann, Parkinson’s disease and upper limb apraxia. *Cortex,**131*, 79–86. 10.1016/j.cortex.2020.05.017 32801083 10.1016/j.cortex.2020.05.017

[41] Liepmann, H. (1920). Apraxie. In H. Brugsch (Ed.), *Ergebnisse der gesamten Medizin* (pp. 516–543). Wien Berlin: Urban & Schwarzenberg.

[42] Randerath, J. (2023). Syndromes of limb apraxia: Developmental and acquired disorders of skilled movements. In G. G. Brown, T. Z. King, K. Y. Haaland, & B. Crosson (Eds.), *APA handbook of neuropsychology: Neurobehavioral disorders and conditions: Accepted science and open questions* (pp. 159–184). American Psychological Association. 10.1037/0000307-008

[43] Hanna-Pladdy, B., Mendoza, J. E., Apostolos, G. T., & Heilman, K. M. (2002). Lateralised motor control: Hemispheric damage and the loss of deftness. *Journal of Neurology, Neurosurgery, and Psychiatry,**73*(5), 574–577. 10.1136/jnnp.73.5.574 12397154 10.1136/jnnp.73.5.574 PMC1738143

[44] Heilman, K. M., Meador, K. J., & Loring, D. W. (2000). Hemispheric asymmetries of limb-kinetic apraxia: A loss of deftness. *Neurology,**55*(4), 523–526. 10.1212/wnl.55.4.523 10953184 10.1212/wnl.55.4.523

[45] Gordon, E. M., Chauvin, R. J., Dosenbach, N. U. F., et al. (2023). A somato-cognitive action network alternates with effector regions in motor cortex. *Nature,**617*(7960), 351–359. 10.1038/s41586-023-05964-2 37076628 10.1038/s41586-023-05964-2 PMC10172144

[46] Nowak, D. A., Grefkes, C., Dafotakis, M., Küst, J., Karbe, H., & Fink, G. R. (2007). Dexterity is impaired at both hands following unilateral subcortical middle cerebral artery stroke. *European Journal of Neuroscience,**25*, 3173–3184. 10.1111/j.1460-9568.2007.05551.x 17561831 10.1111/j.1460-9568.2007.05551.x

[47] Goldenberg, G., Münsinger, U., & Karnath, H. O. (2009). Severity of neglect predicts accuracy of imitation in patients with right hemisphere lesions. *Neuropsychologia,**47*(13), 2948–2952. 10.1016/j.neuropsychologia.2009.06.024 19573545 10.1016/j.neuropsychologia.2009.06.024

[48] Latarnik, S., Wirth, K., Held, A., Kalbe, E., Kessler, J., Saliger, J., Karbe, H., Fink, G. R., & Weiss, P. H. (2020). Prevalence and characteristics of apraxic deficits after left and right hemisphere stroke. *Fortschritte der Neurologie-Psychiatrie,**88*(4), 232–240. 10.1055/a-1082-6501 32325517 10.1055/a-1082-6501

[49] Dressing, A., Martin, M., Beume, L. A., Kuemmerer, D., Urbach, H., Kaller, C. P., Weiller, C., & Rijntjes, M. (2020). The correlation between apraxia and neglect in the right hemisphere: A voxel-based lesion-symptom mapping study in 138 acute stroke patients. *Cortex; A Journal Devoted to the Study of the Nervous System and Behavior,**132*, 166–179. 10.1016/j.cortex.2020.07.017 32987240 10.1016/j.cortex.2020.07.017

