# Supplementary figures and images for: The role of the left primary motor cortex in apraxia
Source: Neurol Res Pract. 2025 Jan 9;7:2. doi: 10.1186/s42466-024-00359-8 (PMC11716253; doi:10.1186/s42466-024-00359-8)

## Slide 1
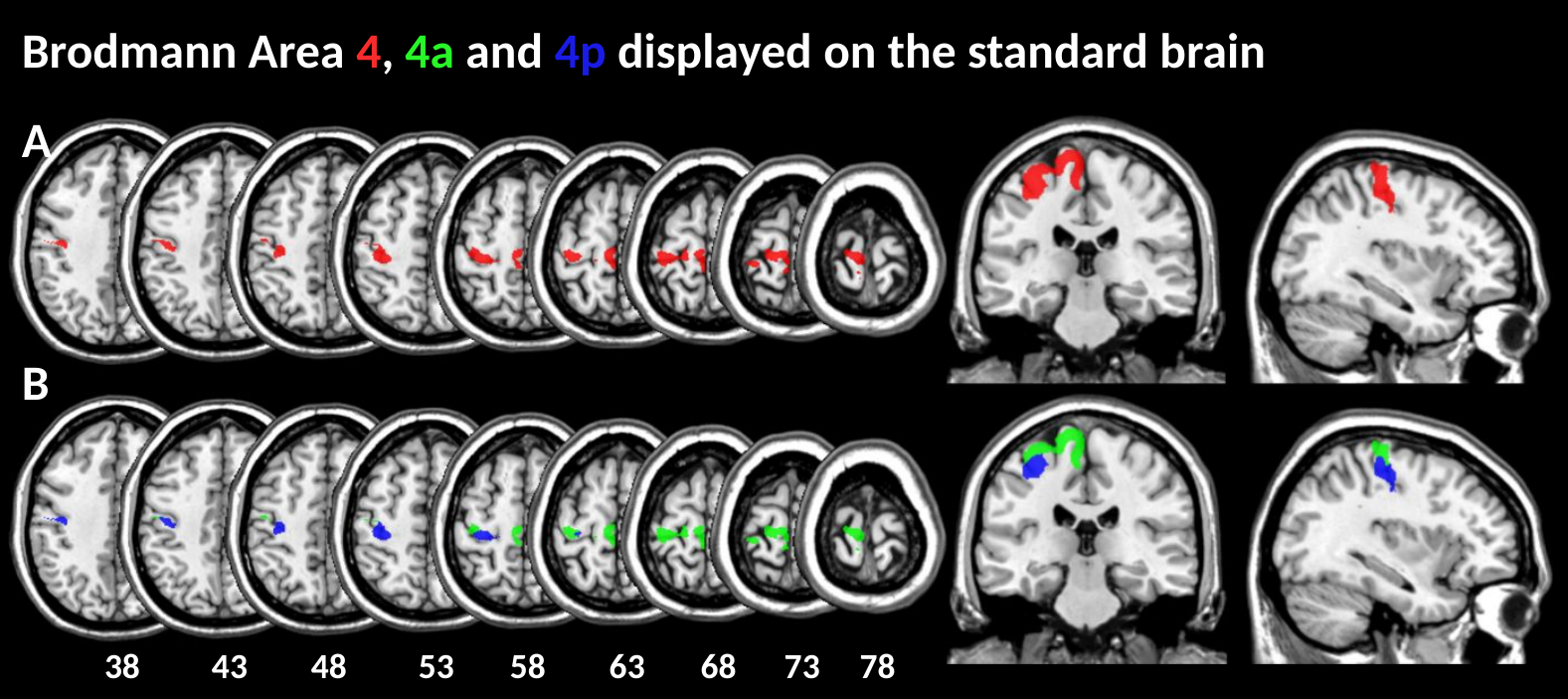

Brodmann Area 4, 4a and 4p displayed on the standard brain
A
B
 38 43 48 53 58 63 68 73 78

Supplement: Supplementary file 1 — Additional file1. Figure S1 – Brodmann Area 4 and its anterior and posterior subareas displayed on the standard brain. A. Brodmann Area 4 (6795 voxels, red); B. Brodmann Areas 4a (4611 voxels, green) and 4p (2184 voxels, blue). The regions of interest (ROIs), taken from the Julich-Brain Atlas (Amunts et al., 2021), are displayed on the standard brain provided by MRIcron. [file 42466_2024_359_MOESM1_ESM.pptx]
